# Hyperglycaemia and Its Risk Factors Among Adults Living With HIV on Follow‐Up at the Hawassa City Administration, Southern Ethiopia: A Cross‐Sectional Study

**DOI:** 10.1002/edm2.70054

**Published:** 2025-04-27

**Authors:** Agete Tadewos Hirigo, Daniel Yilma, Ayalew Astatkie, Zelalem Debebe

**Affiliations:** ^1^ School of Medical Laboratory Science College of Medicine Health Sciences, Hawassa University Hawassa Ethiopia; ^2^ Center for Food Science and Nutrition Addis Ababa University Addis Ababa Ethiopia; ^3^ Department of Internal Medicine College of Public Health and Medical Sciences, Jimma University Jimma Ethiopia; ^4^ Clinical Trial Unit Jimma University Jimma Ethiopia; ^5^ School of Public Health College of Medicine and Health Sciences, Hawassa University Hawassa Ethiopia

**Keywords:** antiretroviral therapy, diabetes mellitus, dolutegravir, HIV, prediabetes, southern Ethiopia, undiagnosed diabetes

## Abstract

**Background:**

Ethiopia implemented the universal test and treat in 2017 and later adopted dolutegravir‐based regimens for people living with HIV (PLWH). However, the impact of these changes on glucose metabolism in Ethiopia remains unclear, highlighting the need for further investigation.

**Methods:**

A cross‐sectional study was conducted in southern Ethiopia from 5 January 2023 to 30 May 2024. We included 443 adult PLWH using systematic random sampling. American Diabetes Association criteria was used to define hyperglycaemia. To identify factors associated with hyperglycaemia, binary logistic regression was used with adjusted odds ratio (AOR) and 95% confidence interval (CI).

**Results:**

Overall prevalence of hyperglycaemia was 24.4% (16.7% prediabetes and 7.7% diabetes mellitus [DM]). Of the participants with DM, 82.3% were newly diagnosed. Significant predictors of hyperglycaemia were age > 50 years (AOR 2.1; 95% CI 1.1–3.9), alcohol intake (AOR 2.1; 95% CI 1.02–4.2), obesity (AOR 3.2; 95% CI 1.3–7.9), high waist–hip ratio (AOR 2.6; 95% CI 1.4–5.05) and LDL‐cholesterol (AOR 2.2; 95% CI 1.02–4.6). While significant predictors of DM were alcohol intake (AOR 3.0; 95% CI 1.1–8.4), co‐morbidity (AOR 2.6; 95% CI 1.1–6.05), high waist circumference (AOR 7.5; 95% CI 1.3–43.3), high waist–hip ratio (AOR 4.1; 95% CI 1.02–16.2) and high triglycerides (AOR 3.2; 95% CI 1.3–7.7). Dolutegravir‐based regimen was not associated with hyperglycaemia.

**Conclusion:**

Hyperglycaemia prevalence among adult PLWH on antiretroviral therapy in southern Ethiopia is rising, with most diabetes cases newly identified. This emphasises the critical need for routine screening to enable early detection, prevention and management.

## Graphical abstract

Hyperglycaemia prevalence among adults living with HIV in southern Ethiopia was 24.4% (16.7% prediabetes, 7.7% diabetes), with 82.3% of diabetes cases newly diagnosed. Predictors included age > 50years, alcohol intake, obesity and dyslipidaemia. Rising rate of hyperglycaemia emphasizes the need for routine screening for early identification, prevention and management.

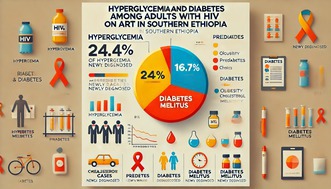

## 1 Introduction

Advancements in combination antiretroviral therapy (ART) have transformed HIV into a manageable chronic condition, significantly increasing the life expectancy of adult people living with HIV (PLWH) [1]. However, the rise in life expectancy has been accompanied by an increasing burden of morbidity and mortality from chronic non‐communicable diseases (NCDs), particularly in low‐ and middle‐income countries (LMICs) [2].

The global prevalence of type 2 diabetes mellitus (T2DM) is projected to reach 11.3% by 2030 and 12.2% by 2040 [3]. In 2021, 464 million adults (9.1%) had impaired glucose tolerance (IGT), and 298 million (5.8%) had impaired fasting glucose (IFG) worldwide [4]. In Africa, a systematic review showed a diabetes prevalence of 5.1% and a prediabetes prevalence of 15.2% in adult PLWH [5]. In sub‐Saharan Africa (SSA), a systematic review conducted between 2000 and 2021 among PLWH reported a prediabetes prevalence of 13.7% (95% confidence interval [CI]: 10.21–17.59) and a diabetes prevalence of 4.8% (95% CI: 4.02–5.59) [5, 6]. In addition, recent cross‐sectional studies have shown a higher prevalence of hyperglycaemia, ranging from 12.8% to 20% [7, 8, 9, 10], with 10.3%–10.9% classified as prediabetes and 2.5%–6.3% as diabetes among PLWH receiving dolutegravir (DTG)‐based first‐line regimens [7, 8]. However, compared to DTG‐based regimens, the prevalence of hyperglycaemia was lower among those who received non‐nucleoside reverse transcriptase inhibitor (NNRTI)‐based regimens, which was 9.4% (with 6.3% prediabetes and 3.1% diabetes) [7].

Older classes of ART drugs, such as protease inhibitors (PIs) and NNRTIs, were used for HIV treatment. However, PIs are associated with metabolic disturbances, including dyslipidemia and insulin resistance, which increase the risk of developing T2DM [11]. Similarly, certain NNRTIs have been implicated in metabolic complications, though to a lesser extent than PIs do [11]. In addition to ART medication effects [12, 13], several other factors contribute to the increased prevalence of hyperglycaemia among PLWH. These include advanced age [12, 14], central obesity [13], hypertension [12, 13], chronic inflammation associated with HIV [15], excessive weight or obesity [16], dyslipidaemia [12, 16] and pulmonary tuberculosis (TB), all of which are associated with insulin resistance [17]. Additional risk factors for hyperglycaemia are certain ethnic backgrounds, a family history of diabetes and male sex [8].

The introduction of newer ART agents, such as integrase strand transfer inhibitors (INSTIs), marked a significant advancement in HIV treatment. Among these, DTG is particularly notable due to its strong antiviral efficacy, high barrier to resistance and better tolerability compared to older drug classes like NNRTIs [18]. In Ethiopia, DTG‐based regimens have been included in HIV treatment protocols since 2018 [19], aligning with WHO guidelines intended to optimise treatment efficacy and improve outcomes for PLWH [18].

Despite its therapeutic advantages, DTG has been associated with potential metabolic side effects including obesity, altered glucose metabolism and insulin resistance [8]. However, the literature on the incidence of hyperglycaemia associated with DTG‐based regimens reveals inconsistent findings. Some studies suggest a potential association between the use of DTG‐based regimens and an elevated risk of hyperglycaemia, possibly influenced by their effects on insulin sensitivity and glucose metabolism [10, 20]. The exact mechanisms behind these effects are not fully understood, as indicated by previous studies [8, 21]. In contrast, other studies have reported comparable metabolic outcomes or found no significant differences between DTG‐based and non‐DTG‐based antiretroviral regimens [22, 23].

Inconsistent findings across studies coupled with varying screening services for PLWH [24] highlight the need for further studies in diverse settings like Ethiopia. Genetic, nutritional and socio‐economic factors may also influence metabolic outcomes. Understanding the burden of hyperglycaemia and identifying modifiable risk factors are crucial for effective prevention and patient care [25]. With limited studies on the prevalence and association of hyperglycaemia with DTG‐based regimens in Ethiopian healthcare settings, conducting further studies to explore this phenomenon is imperative to enhance the quality of care for this population. Thus, this study aimed to determine the prevalence and predictors of hyperglycaemia among adult PLWH on first‐line ART for at least 12 months following the implementation of the test‐and‐treat strategy in Ethiopia.

## 2 Methods

### 2.1 Study Design, Setting and Population

This cross‐sectional study was conducted from 5 January 2023 to 30 May 2024 among adult PLWH receiving follow‐up care at health facilities in Hawassa City administration. The city administration is located in the Sidama Regional State of southern Ethiopia, located approximately 275 km from Ethiopia's capital, Addis Ababa. During the period of this study, eight healthcare facilities within the city administration offering ART services to more than 6300 PLWH. However, participants from two facilities were not included in the study due to personal preferences.

### 2.2 Inclusion and Exclusion Criteria

This study included adults aged 18–65 years who initiated first‐line ART after Ethiopia implemented the test‐and‐treat strategy in February 2017. Eligible participants were those who had received first‐line ART for at least 12 months, achieved a viral load (HIV‐ribonucleic acid [RNA]) level of < 1000 copies/ml and demonstrated at least fair adherence as described in the national HIV management guidelines [19]. The study also included individuals who initiated first‐line ART with two nucleos(t)ide reverse transcriptase inhibitors (N(t)RTIs) as the backbone, combined with one NNRTI, and were either maintained on NNRTI‐based regimens or later switched to a DTG‐based regimen with the same backbone. Moreover, the study also included individuals who initiated and were maintained on two N(t)RTIs with a DTG‐based regimen. However, we deliberately excluded participants on ART who had communication difficulties, experienced multiple treatment switches, were pregnant or lactating, were using antipsychotics or had critical illness, physical disabilities, liver disease or end‐stage renal failure.

### 2.3 Sample Size Determination and Sampling Technique

The sample size was determined using the single population proportion formula and calculated with Epi Info version 7.2 software, assuming a 95% confidence interval (CI), a significance level (α) of 5%, a margin of error of 0.05 and an anticipated DM incidence of 16% among PLWH on ART in Ethiopia [26]. The initial calculated sample size was 229, with a 10% adjustment for non‐response. This study was part of a broader NCDs project with multiple objectives. The sample size of 279 determined based on a 23.9% prevalence rate for one of the NCDs [27] was larger than that calculated for hyperglycaemia, ensuring better representativeness for the overall evaluation of NCDs. Accounting for a design effect of 1.5 and an anticipated 10% non‐response rate, we calculated the final sample size to be 465. Allocation was then based on the number of PLWH on first‐line regimens at each facility. We calculated the sampling interval for each facility by dividing the number of PLWH on first‐line ART by the allocated sample size. Participants were selected using a systematic random sampling method within each health facility.

### 2.4 Data Collection and Procedure

Socio‐demographic, socio‐economic, clinical and other relevant data were collected using an interviewer‐administered structured questionnaire. Trained nurses working in the respective ART clinics conducted the interviews and measurements. They also extracted relevant clinical data from participants' medical records.

### 2.5 Variables and Measurement

#### 2.5.1 Dependent Variable

In this study, we defined hyperglycaemia among adults with HIV as the primary outcome variable, using a fasting blood glucose (FBG) level of ≥ 100 mg/dL, which includes both prediabetes and T2DM. We classified hyperglycaemia according to the guidelines from the American Diabetes Association (ADA), which have been adopted in the Clinical and Programmatic Management guidelines of Major NCDs in Ethiopia [28, 29]. Diabetes was diagnosed based on FBG ≥ 126 mg/dL, self‐reported history and antidiabetic medication use or confirmed from medical records. Undiagnosed diabetes or newly diagnosed DM during this study was defined as FBG ≥ 126 mg/dL, confirmed by a repeat test of the same sample. Prediabetes (IFG) was defined by FBG levels between 100 and 125 mg/dL.

#### 2.5.2 Independent Variables

This study examined various predictors across multiple domains. Patient‐related factors included sex, age, occupation, economic status, marital status, residence and education, along with anthropometric measurements such as weight, body mass index (BMI), waist and hip circumference (HC), waist–hip ratio (WHR), waist–height ratio (WHtR) and visceral fat level. Clinical and HIV‐related factors considered were co‐morbid conditions, baseline WHO disease stage, baseline functional status, opportunistic infections, duration since HIV diagnosis and mental health issues (anxiety, depression, insomnia, memory). Behavioural factors such as alcohol, cigarette and khat use as well as diet and physical activity were also assessed. Finally, treatment‐related factors, including ART regimen type, treatment switches and duration since ART initiation, were evaluated as potential predictors.

Height was determined to the nearest 0.1cm using a stadiometer (Seca, Germany) with participants standing in the Frankfurt plane after removing shoes, hats and hair ornaments. The Omron Body Composition Monitor and Scale (Omron HBF‐514C; Omron Healthcare Co. Ltd.) was utilised to assess participants' body weight, BMI and visceral fat level. This device employs bioelectrical impedance analysis (BIA) [30]. To measure body composition, each participant's age, height (in cm) and sex were entered into the device before they were instructed to step barefoot onto the scale. Data collectors instructed each participant to wear light clothes and stand barefoot on the scale, holding the display unit with both hands and extending their arms parallel to the floor while maintaining an upright posture. The device measures body composition by transmitting a painless, low‐level electrical current through various body tissues. It directly measures weight and VF levels, whereas BMI was calculated via initially entered height information. The BMI of the participants was subsequently categorised as underweight (< 18.5 kg/m^2^), normal (18.5–24.99 kg/m^2^), overweight (25.0–29.99 kg/m^2^) or obese (≥ 30 kg/m^2^) [31]. The visceral fat level was assessed in accordance with the Omron Healthcare manual [30].

WC and HC were measured to the nearest 0.1 cm. WC was measured in a horizontal plane, midway between the iliac crest and the costal margin, at the end of a normal expiration. While the HC was measured at the level of the greater trochanter via a non‐stretchable tape measure [32]. The WHR and WHtR were then calculated by dividing WC by HC and WC by height respectively. Abdominal obesity was classified as a WC ≥ 94 cm for males and ≥ 80 cm for females or a WHR ≥ 0.90 for men and ≥ 0.85 for women. Additionally, a WHtR ≥ 0.5 was considered indicative of an increased risk of metabolic complications [33].

Participants' blood pressure (BP) and heart rate were assessed via a digital blood pressure monitor (Heuer digital sphygmomanometer) after 10 min resting in the data collection room. Two BP readings were taken at a 5‐min interval. If the difference was greater than 5 mmHg, a third reading was obtained, and the average of the last two readings was recorded to determine the participant's BP status [34]. The International Physical Activity Questionnaire short form (IPAQ‐SF) was employed to assess participants' physical activity levels. Activity levels were classified based on metabolic equivalents of task in minutes per week (MET‐min/wk) as low (< 600 MET‐min/wk), moderate (600 to < 3000 MET‐min/wk) or high (≥ 3000 MET‐min/wk) [35]. Participants were asked about their weekly frequency of fruit and vegetable (FAV) consumption, including the number of servings consumed on a typical day. Adequate FAV intake is defined by the WHO as the intake of 400–500 g/day, or approximately 5 servings (80 g each) [36]. Moreover, substance use was categorised as follows: participants who chewed khat, smoked cigarettes or consumed alcohol within the 30 days prior to the study were considered current users. Those with a history of using khat, cigarettes or alcohol at any point in their lifetime but who had not engaged in these behaviours in the past 30 days were classified as former users [37]. Furthermore, anxiety and depression were assessed via a validated questionnaire (HADS) in the Amharic language for the Ethiopian population. It has seven questions each, and the responses are rated on a 4‐point Likert scale, with a score of ≥ 8 indicating the presence of anxiety or depression [38, 39].

### 2.6 Blood Sample Collection and Laboratory Analysis

Upon arrival at the ART clinic, all participants were asked whether they had fasted overnight (8–12 h), with the exception of water. Participants who had fasted were asked to provide a blood sample, while those who had eaten breakfast were advised to fast overnight (8–12 h) and return either the next day or at their next ART clinic visit to provide a sample. Then, overnight fasting blood was drawn from each participant in two separate test tubes: one in a gel‐based serum separator tube with a clot activator and the other in an ethylene–diamine–tetraacetic acid (EDTA) anticoagulant containing tube. Both sample tubes were labelled with the participant's unique identification code. After collection, whole blood samples were kept at room temperature for a maximum of 20 min to allow clot formation and avoid glucose consumption through glycolysis [40, 41, 42, 43]. The samples were promptly centrifuged at 3000 rpm for 8–10 min, allowing serum separation through a gel barrier. Subsequently the samples were analysed for FBG and lipid profiles, whereas EDTA blood was used for determination of glycated haemoglobin (HbA1c). If there was a delay in laboratory analysis, serum samples were promptly transferred to Nunc tubes labelled with participants' unique codes and stored at −20°C until analysis. Blood collection for HbA1c testing was prioritised for participants newly diagnosed with diabetes (FBG ≥ 126 mg/dL) due to the limited availability of reagents. The remaining HbA1c samples were stored in the deep fridge at −70°C. Lipid profiles were analysed using enzymatic colorimetric methods, FBG was assessed via the hexokinase UV method and HbA1c was measured using turbidimetric inhibition immunoassay (TINIA) on a Cobas 6000 series analyser with Roche reagents (Roche, Germany).

### 2.7 Data Quality Management

The questionnaire was pretested on 46 similar adult participants at Leku General Hospital in the Sidama region and revised based on the feedback received. The questionnaire was checked daily to ensure consistency, completeness, clarity and accuracy of data collection. Nurses in the ART clinics received training on data collection and physical measurements. In addition, weight scales were calibrated daily against a standard calibrating tool before data collection. The physical measurements included at least two readings per participant and were averaged for reliability. Additionally, laboratory procedures were rigorously managed by Lab technologists in accordance with standard operating procedures (SOPs). Daily assessment of chemistry analyser was done performed to ensure precision of technical performance and reagent quality by running lyophilised quality control (QC) samples before the study samples. Furthermore, the principal investigator closely supervised the overall process to ensure data quality.

### 2.8 Statistical Analysis

The data were entered into EpiData version 3.1 and exported to IBM SPSS version 27.0 for statistical analysis. Descriptive statistics, including means with standard deviation (SD), medians with interquartile ranges (IQRs) and percentages were computed to summarise participant characteristics. The normality of the data was assessed via histograms, Q–Q plots and the Kolmogorov–Smirnov test (K‐S). The facility–level effect was insignificant, as indicated by an intra‐class correlation coefficient (ICC) of 0.026, showing minimal variation between health facilities. Therefore, we opted to use a binary logistic regression model to examine the associations between hyperglycaemia and predictors. Independent variables with a *p* < 0.25 from the bivariate analysis were considered for inclusion in the multivariate analysis, along with variables identified in previous studies as risk factors for hyperglycaemia, regardless of their individual significance level in the bivariate analysis. Multi‐collinearity of candidate variables was assessed, with those having a variance inflation factor (VIF) < 10 included in the final model. Model fit was evaluated using the Hosmer–Lemeshow test, where a *p* > 0.05 indicated a good fit. Adjusted odds ratio (AOR) and 95% CIs were used to measure the strength of associations between risk factors and outcomes.

## 3 Results

### 3.1 Socio‐Demographic Features of the Study Participants

Out of 465 eligible adults, 443 participated in the study, with a response rate of 95.3%. More than half of the participants (*n* = 259, 58.5%) were women, with a mean age of 40.9 (SD, 9.6) years. The majority of the participants (68.4%) were 30–49 years old. Half of the participants were married and more than 41% had a monthly income of ≤ 2000 Ethiopian birr. In terms of education, 11.3% of participants had no formal education, while 57.1% had at least a secondary level education (Table 1).

**TABLE 1:** Socio‐demographic features of the study population.

Variables	Category	Frequency	%
Sex	Female	259	58.5
	Male	184	41.5
Age	20–30 years	67	15.1
	31–40 years	172	38.8
	41–50 years	134	30.2
	50^+^ years	70	15.8
Residence	Rural	41	9.3
	Urban	402	90.7
Marital status	Single	47	10.6
	Married	222	50.1
	Divorced	102	23.0
	Widow/widower	72	16.3
Educational status	Not educated	50	11.3
	Informal	1	0.2
	Primary level	139	31.4
	Secondary level	128	28.9
	At least college	125	28.2
Occupation	Employed	190	42.9
	Farmers	10	2.3
	Merchants	89	20.1
	Unemployed	154	34.8
Monthly income in Ethiopian birr	< 5000 birr	276	62.3
	5000–10,000 birr	139	31.4
	> 10,000 birr	28	6.3

### 3.2 Physical Measurements, Behavioural Characteristics, Physical Activity Levels and Dietary Patterns of the Study Participants

The median time since HIV diagnosis was 4.2 (IQR, 2.3–6.1) years, while the mean duration on ART was 3.8 (SD, 1.9) years. The mean BMI of the study participants was 24.7 (SD, 4.8) kg/m^2^. About 44.3% of participants had a BMI of ≥ 25 kg/m^2^, with 31.2% classified as overweight and 13.1% as obese. Regarding self‐reported substance use, 58.2% of participants reported ever‐consuming alcohol, 37.7% had chewed khat and 14.5% had smoked cigarettes. In terms of physical activity, 32.3% of participants had inadequate physical activity, while 55.1% engaged in moderate physical activity. Only 10 (2.3%) and 15 (3.4%) respondents reported daily consumption of fruits and vegetables, respectively, while 35.4% did not consume any fruits and 9.9% did not consume any vegetables in the week prior to the study (Table S1).

### 3.3 Duration and Type of ART Regimens Used by the Study Participants

Initially, 218 (49.2%) participants began treatment with NNRTI‐based first‐line regimens; of these, 203 (93.1%) were subsequently transitioned to DTG‐based first‐line regimens. In addition, 225 (50.8%) participants initiated therapy with a DTG‐based regimen and maintained it. At the time of the study, over 95% of the participants had been exposed to a DTG‐based regimen, either as their initial treatment or following a switch.

### 3.4 Overall Prevalence of Hyperglycaemia Among the Study Participants

**FIGURE 1:**
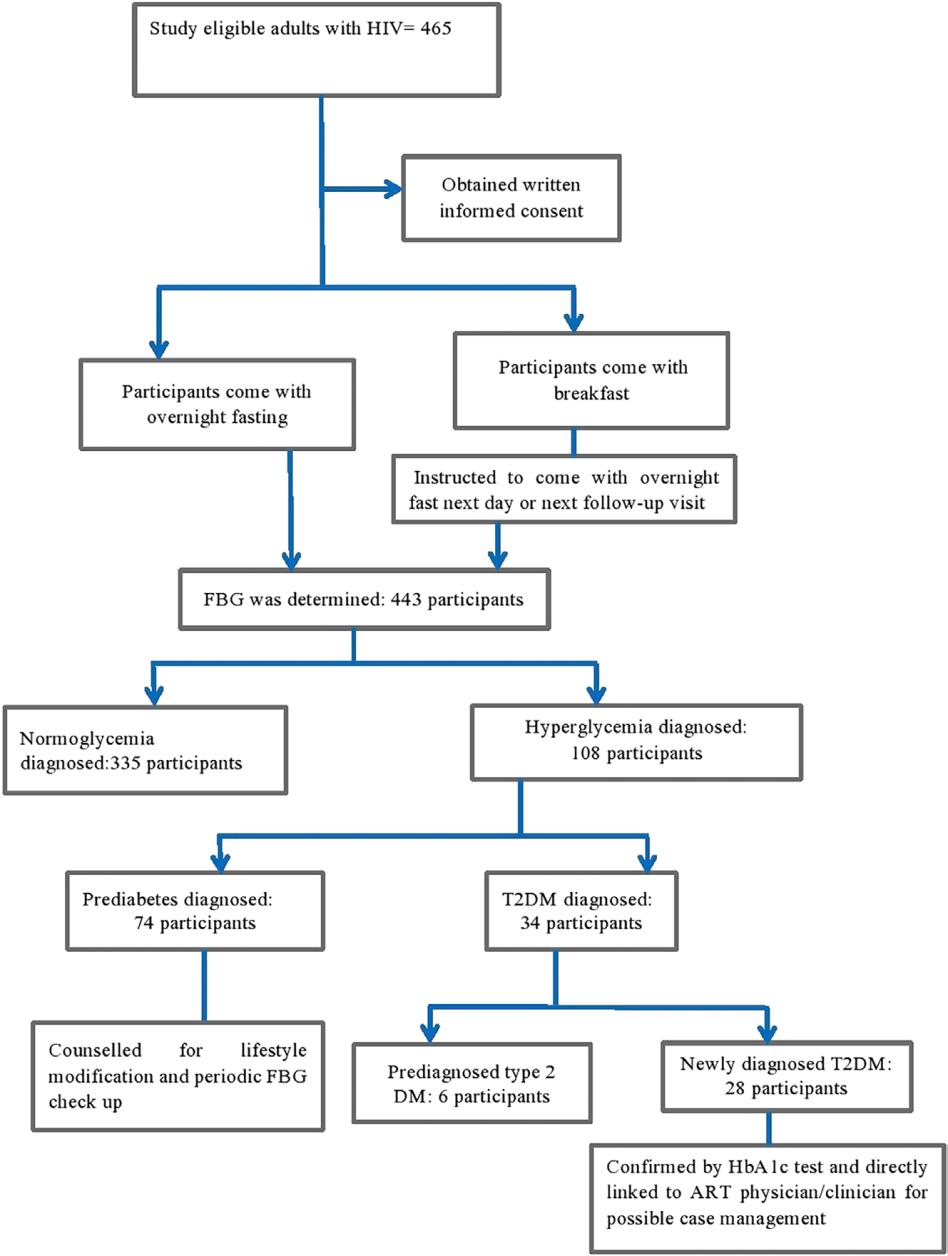
Diagnostic categorisation and prevalence of hyperglycaemia among the study population.

**FIGURE 2:**
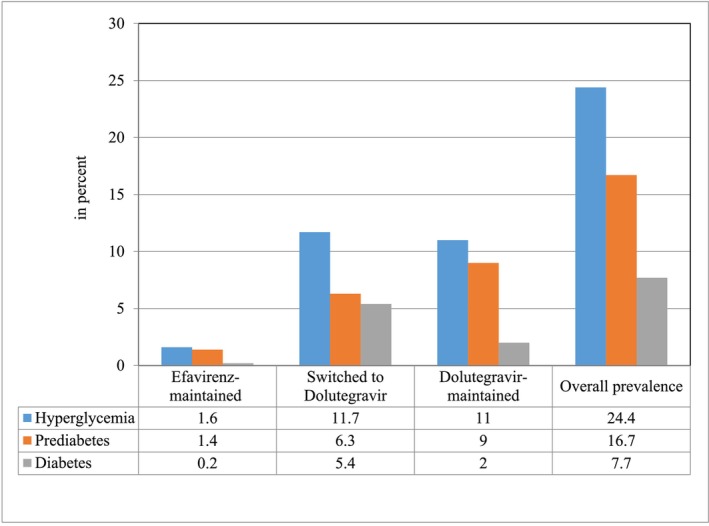
Prevalence of overall hyperglycaemia, prediabetes and diabetes mellitus among the study population.

The overall prevalence of hyperglycaemia was 24.4% (95% CI: 20.3–28.7). In men, it was 27.2% (95% CI: 21.2–33.7), while in women, it was 22.4% (95% CI: 17.0–27.4). Hyperglycaemic participants were older than those with normoglycaemia, with a mean age of 44.4 (SD, 9.3) years compared to 39.8 (SD, 9.5) years in the normoglycaemic group (*p* < 0.001). The prevalence of prediabetes and T2DM was 16.7% (95% CI: 13.1–20.3) and 7.7% (95% CI: 5.2–10.4) respectively. The prevalence of prediabetes among women was 18.5% (95% CI: 13.6–23.9), while 8.7% (95% CI: 4.9–13.0) had T2DM. In men, 15.4% (95% CI: 11.2–20.1) had prediabetes and 6.9% (95% CI: 3.9–10.0) were diagnosed with T2DM. Among the 34 participants diagnosed with T2DM, 28 (82.35%) were newly identified during this study (Figures 1 and 2).

### 3.5 Factors Associated With Overall Hyperglycaemia and Diabetes Among the Study Population

After adjusting for confounding factors, individuals over 50 years of age had twice the odds of hyperglycaemia compared to those aged 50 or younger (AOR: 2.1; 95% CI: 1.1–3.9). The odds of hyperglycaemia were also twice as high among individuals who currently drink alcohol compared to those who do not (AOR: 2.1; 95% CI: 1.02–4.2). Individuals with a high WHR had 2.6 times greater odds of hyperglycaemia than those with a normal WHR (AOR: 2.6; 95% CI: 1.4–5.05). Moreover, individuals with a BMI ≥ 30 kg/m^2^ had three times the odds of hyperglycaemia compared to those with a BMI < 25 kg/m^2^ (AOR: 3.2; 95% CI: 1.3–7.9) (Table 2).

**TABLE 2:** Factors associated with hyperglycaemia among the study population.

Variables	Category	Total *n* = 443	Hyperglycaemia, *n* (%)		COR (95% CI)	*p*	AOR (95% CI)	*p*
			Yes (*n* = 108)	No (*n* = 335)				
Age	≤ 50 years	373 (84.2)	80 (74.1)	293 (87.5)	1.00		1.00	
	> 50 years	70 (15.8)	28 (25.9)	42 (12.5)	2.4 (1.4–4.2)	< 0.001	2.1 (1.1–3.9)	0.021
Sex	Female	259 (58.5)	58 (53.7)	201 (60.0)	1.00		1.00	
	Male	184 (41.5)	50 (46.3)	134 (40.0)	1.3 (0.83–2.0)	0.249	0.98 (0.51–1.9)	0.948
Time since HIV diagnosis, years	Median (IQR)	4.2 (2.3–6.2)	4.4 (2.4–6.3)	4.2 (2.2–6)	1.05 (0.98–1.1)	0.169	0.99 (0.9–1.08)	0.816
Family history of diabetes	No	360 (81.3)	83 (76.9)	277 (82.7)	1.00		1.00	
	Yes	83 (18.7)	25 (23.1)	58 (17.3)	1.4 (0.85–2.4)	0.178	1.1 (0.63–2.05)	0.664
Treatment groups	Maintained on efavirenz	15 (3.4)	7 (6.5)	8 (2.4)	1.00		1.00	
	Switched to dolutegravir	203 (45.8)	52 (48.1)	151 (45.1)	0.39 (0.14–1.14)	0.085	0.46 (0.14–1.5)	0.204
	Maintained on dolutegravir	225 (50.8)	49 (45.4)	176 (52.5)	0.32 (0.11–0.92)	0.035	041 (0.12–1.4)	0.161
Received prophylaxis for OIs	No	151 (34.1)	42 (38.9)	109 (32.5)	1.00		1.00	
	Yes	292 (65.9)	66 (61.1)	226 (67.5)	0.76 (0.48–1.2)	0.227	0.69 (0.41–1.2)	0.163
Waist circumference	Normal	218 (49.2)	33 (30.6)	185 (55.2)	1.00		1.00	
	High	225 (50.8)	75 (69.4)	150 (44.8)	2.8 (1.7–4.4)	< 0.001	1.3 (0.53–3.0)	0.601
Waist–hip ratio	Normal	183 (41.3)	23 (21.3)	160 (47.8)	1.00		1.00	
	High	260 (58.7)	85 (78.7)	175 (52.2)	3.4 (2.0–5.6)	< 0.001	2.6 (1.4–5.05)	0.003
OIs other than TB	No	358 (80.8)	92 (85.2)	266 (79.4)	1.00		1.00	
	Yes	85 (19.2)	16 (14.8)	69 (20.6)	0.67 (0.37–1.2)	0.187	0.75 (0.39–1.4)	0.391
Alcohol intake	Never	185 (41.8)	37 (34.3)	148 (44.2)	1.00		1.00	
	Former	172 (38.8)	40 (37)	132 (39.4)	1.2 (0.73–2.0)	0.455	0.99 (0.54–1.8)	0.998
	Current	86 (19.4)	31 (28.7)	55 (16.4)	2.2 (1.3–4.0)	0.005	2.1 (1.02–4.2)	0.044
LDL‐cholesterol	< 130 mg/dL	403 (91.0)	91 (84.3)	312 (93.1)	1.00		1.00	
	≥ 130 mg/dL	40 (9.0)	17 (15.7)	23 (6.9)	2.5 (1.3–4.9)	0.006	2.2 (1.02–4.6)	0.043
Days of fruit intake/week	Median (IQR)	1.0 (0–3)	2.0 (0–3)	1.0 (0–3)	1.1 (1.01–1.3)	0.033	1.1 (0.95–1.3)	0.197
Days of vegetable intake	≥ 4 days/week	88 (19.9)	17 (15.7)	71 (21.2)	1.00		1.00	
	< 4 days/week	355 (80.1)	91 (84.3)	264 (78.8)	1.4 (0.81–2.6)	0.219	1.8 (0.94–3.5)	0.074
Waist–height ratio	< 0.5	156 (35.2)	22 (20.4)	134 (40.0)	1.00		1.00	
	≥ 0.5	287 (64.8)	86 (79.6)	201 (60.0)	2.6 (1.5–4.4)	< 0.001	0.64 (0.26–1.6)	0.340
Body mass index	< 25 kg/m^2^	247 (55.8)	40 (37.0)	207 (61.8)	1.00		1.00	
	25–29.9 kg/m^2^	138 (31.2)	43 (39.8)	95 (28.3)	2.3 (1.4–3.8)	< 0.001	1.7 (0.79–3.5)	0.176
	≥ 30 kg/m^2^	58 (13.1)	25 (23.1)	33 (9.9)	3.9 (2.1–7.3)	< 0.001	3.2 (1.3–7.9)	0.012
Monthly income in Ethiopian birr	< 5000	276 (62.3)	56 (51.9)	220 (65.7)	1.00		1.00	
	5000–10,000	139 (31.4)	43 (39.8)	96 (28.7)	1.8 (1.1–2.8)	0.017	1.1 (0.66–1.9)	0.651
	> 10,000	28 (6.3)	9 (8.3)	19 (5.7)	1.9 (0.8–4.3)	0.150	0.91 (0.32–2.6)	0.651
Physical activity performance status	High	56 (12.6)	8 (7.4)	48 (14.3)	1.00		1.00	
	Low	143 (32.3)	45 (41.7)	98 (29.3)	2.7 (1.2–6.3)	0.016	1.3 (0.52–3.4)	0.867
	Moderate	244 (55.1)	55 (50.9)	189 (56.4)	1.7 (0.8–3.9)	0.176	1.3 (0.55–3.1)	0.529

*Note:* High waist circumference: ≥ 94 cm in men and ≥ 80 cm in women; high waist–hip ratio: ≥ 0.90 in men and ≥ 0.85 in women.

Abbreviations: AOR, adjusted odds ratio; COR, crude odds ratio; dL, decilitre; IQR, interquartile range; kg, kilogram; LDL, low‐density lipoprotein; m^2^, square meter; mg, milligram; OIs, opportunistic infections; TB, tuberculosis.

The odds of developing T2DM was three times higher among individuals who currently drink alcohol than those who do not (AOR: 3.0; 95% CI: 1.1–8.4). Individuals with HIV and additional co‐morbidities had 2.6 times higher odds of developing T2DM compared to those without co‐morbidities (AOR: 2.6; 95% CI: 1.1–6.05). Additionally, individuals with a high WC had nearly eight times higher odds of DM compared to those with a normal WC (AOR: 7.5; 95% CI: 1.3–43.3). The odds of DM were three times greater among individuals with high triglycerides than those with normal triglyceride levels (AOR: 3.2; 95% CI: 1.3–7.7). However, DTG‐based ART regimens did not show a significant association with T2DM when compared to EFV‐based regimens (Table 3). Statistically significant interaction effects were observed between treatment regimens and age > 50 years, as well as between treatment regimens and BMI on hyperglycaemia (Table S2). Furthermore, sex‐stratified analyses revealed significant interaction effects between DTG‐based regimens and age > 50, overweight status and high triglyceride levels on hyperglycaemia (Table S3).

**TABLE 3:** Factors associated with diabetes mellitus among the study population.

Variables	Category	Total (*n* = 443)	Diabetes mellitus, *n* (%)		COR (95% CI)	*p*	AOR (95% CI)	*p*
			Yes (*n* = 34)	No (*n* = 409)				
Age	≤ 50 years	373 (84.2)	25 (73.5)	348 (85.1)	1.00		1.00	
	> 50 years	70 (15.8)	9 (26.5)	61 (14.9)	2.0 (0.9–4.6)	0.081	1.4 (0.50–3.9)	0.517
Treatment duration	< 5 years	283 (63.9)	16 (47.1)	267 (65.3)	1.00		1.00	
	≥ 5 years	160 (36.1)	18 (52.9)	142 (34.7)	2.1 (1.05–4.2)	0.037	1.1 (0.37–3.1)	0.894
Time since HIV diagnosis, years	Median (IQR)	4.2 (2.3–6.2)	5.4 (3–6.7)	4 (2.2–6.0)	1.1 (1.01–1.2	0.026	1.01 (0.9–1.2)	0.875
Treatment groups	Maintained on efavirenz	15 (3.4)	1 (2.9)	14 (3.4)	1.00		1.00	
	Switched to dolutegravir	203 (45.8)	24 (70.6)	179 (43.8)	1.9 (0.2–14.9)	0.552	2.1 (0.2–22.7)	0.523
	Maintained on dolutegravir	225 (50.8)	9 (26.5)	216 (52.8)	0.6 (0.07–4.9)	0.621	0.69 (0.06–8.3)	0.771
Waist circumference	Normal	218 (49.2)	6 (17.6)	212 (51.8)	1.00		1.00	
	High	225 (50.8)	28 (82.4)	197 (48.2)	5 (2.0–12.4)	< 0.001	7.5 (1.3–43.3)	0.024
Waist–hip ratio	Normal	183 (41.3)	4 (11.8)	179 (43.8)	1.00		1.00	
	High	260 (58.7)	30 (88.2)	230 (56.2)	5.8 (2.0–17)	< 0.001	4.1 (1.02–16.2)	0.047
Anxiety/depression	No	425 (95.9)	31 (91.2)	394 (96.3)	1.00		1.00	
	Yes	18 (4.1)	3 (8.8)	15 (3.7)	2.5 (0.70–9.2)	0.157	2.9 (0.58–14.6)	0.192
Alcohol intake	Never	185 (41.8)	12 (35.3)	173 (42.3)	1.00		1.00	
	Former	172 (38.8)	8 (23.5)	164 (40.1)	0.7 (0.28–1.8)	0.453	0.54 (0.19–1.5)	0.251
	Current	86 (19.4)	14 (41.2)	72 (17.6)	2.8 (1.2–6.3)	0.014	3.0 (1.1–8.4)	0.037
Co‐morbidity with HIV*	No	357 (80.6)	20 (58.8)	337 (82.4)	1.00		1.00	
	Yes	86 (19.4)	14 (41.2)	72 (17.6)	3.3 (1.6–6.8)	< 0.001	2.6 (1.1–6.05)	0.03
Triglycerides	< 150 mg/dL	292 (65.9)	13 (38.2)	279 (68.2)	1.00		1.00	
	≥ 150 mg/dL	151 (34.1)	21 (61.8)	130 (31.8)	3.5 (1.7–7.1)	< 0.001	3.2 (1.3–7.7)	0.09
Cholesterol total	< 200 mg/dL	386 (97.1)	27 (79.4)	359 (87.8)	1.00		1.00	
	≥ 200 mg/dL	57 (12.9)	7 (20.6)	50 (12.2)	1.8 (0.77–4.5)	0.168	0.83 (0.28–2.4)	0.833
Days of vegetable intake	≥ 4 days/week	355 (80.1)	30 (88.2)	325 (79.5)	1.00		1.00	
	< 4 days/week	88 (19.9)	4 (11.8)	84 (20.5)	1.9 (0.66–5.6)	0.226	2.5 (0.77–8.5)	0.126
Waist–height ratio	< 0.5	156 (35.2)	5 (14.7)	141 (36.9)	1.00		1.00	
	≥ 0.5	287 (64.8)	29 (85.3)	258 (63.1)	3.4 (1.3–8.9)	0.014	0.19 (0.02–1.4)	0.105
Body mass index	< 25 kg/m^2^	247 (55.8)	12 (35.3)	235 (57.5)	1.00		1.00	
	25–29.9 kg/m^2^	138 (31.2)	16 (47.1)	122 (29.8)	2.6 (1.2–5.6)	0.018	0.96 (0.3–3.2)	0.947
	≥ 30 kg/m^2^	58 (13.1)	6 (17.6)	52 (12.7)	2.3 (0.81–6.3)	0.119	1.2 (0.2–6.7)	0.864
Visceral fat level	Median (IQR)	4.2 (2.3–6.2)	8.3 (4.7–13)	5.4 (3–6.7)	1.1 (1.02–1.2)	0.013	0.94 (0.77–1.1)	0.581
Physical activity performance status	High	56 (12.6)	2 (5.9)	54 (13.2)	1.00		1.00	
	Insufficient	143 (32.3)	15 (44.1)	128 (31.3)	3.2 (0.7–14.3)	0.135	2.1 (0.38–12.1)	0.390
	Moderate	244 (55.1)	17 (50)	227 (55.5)	2.0 (0.45–9.0)	0.356	2 (0.37–11.2)	0.413

*Note:* High waist circumference: ≥ 94 cm in men and ≥ 80 cm in women; high waist–hip ratio: ≥ 0.90 in men and ≥ 0.85 in women.

Abbreviations: AOR, adjusted odds ratio; COR, crude odds ratio; dL, decilitre; IQR, interquartile range; kg, kilogram; m^2^, square meter; mg, milligram.

* Coexistence of hypertension, cancer and or other chronic diseaes with HIV.

## 4 Discussion

With limited research on the prevalence of hyperglycaemia and its association with first‐line regimens, particularly those containing DTG, coupled with inconsistent findings, further studies are needed to address its extent and determinants in order to improve care and outcomes among PLWH. This cross‐sectional study describes the prevalence and associated factors of hyperglycaemia among adult PLWH on first‐line ART for at least 12 months following the implementation of the test‐and‐treat strategy in southern Ethiopia.

The prevalence of hyperglycaemia among adult PLWH on ART in this study was 24.4%. This finding is aligned with the previous studies that were conducted in Ethiopia and Africa, showing hyperglycaemia prevalence rates between 18.5% and 28% [5, 8, 44]. However, the finding is higher than the hyperglycaemia rates reported in several studies among individuals on INSTI based and other ART regimens across Ethiopia: 7.9% in central Ethiopia [45], 14.7% in Tigray [46], 17.2% in Amhara [7] and 17.2% in Eastern Ethiopia [47]. Similarly, it is higher than the rates reported elsewhere, such as 15% in Zambia [48], 8.3% in Georgia [49] and 14.4% in the United States [50]. These inconsistencies may arise from differences in treatment duration, hyperglycaemia cutoffs (100 mg/dL vs. 110 mg/dL) and the varying effects of older versus newer ART regimens on glucose metabolism. Moreover, the finding is higher than the 12.4% prevalence reported in the 2015 National NCDs STEPS Survey of the general population in Ethiopia using ADA criteria and 7.1% using the WHO criteria [51]. This suggests that the factors contributing to hyperglycaemia in PLWH who are receiving ART may differ from those in the general population and may be influenced by factors such as HIV infection itself, HIV duration, immunosuppression and the effects of antiretroviral medication [52].

We found substantial rates of prediabetes (16.7%) and T2DM (7.7%) in this study. This is higher than the rate estimates reported for the general adult population in Ethiopia, which is 3.3% according to the IDF in 2021 [53] and 3.8% according to the WHO in 2016 [54]. Additionally, it is higher than the 2015 National NCDs STEPS Survey report, which revealed 9.1% for prediabetes and 3.3% for diabetes among adults in Ethiopia [51]. The differences in prediabetes and diabetes prevalence between our study population and the general population may be influenced by several factors. PLWH may be more vulnerable to health complications due to a compromised immune system, potentially leading to higher rates of prediabetes and T2DM. Additionally, medications used in HIV treatments have the potential to alter metabolism, influencing glucose regulation and increasing diabetes risk compared with the general population. Our findings also revealed higher rates of prediabetes and T2DM compared to previous studies conducted in PLWH: 14.2% prediabetes and 5% T2DM in Kenya [13], 13% prediabetes and 1.4% T2DM in the United States [50], 5.1% DM across Africa [5] and 10% prediabetes and 5% T2DM in Zambia [48]. The higher rates of prediabetes and T2DM observed in our study compared to the described studies may be attributable to several factors. Factors include the longer duration of DTG use in participants, regional and demographic variations in healthcare access [8], differences in diagnostic criteria [8] and disparities in healthcare infrastructure and diabetes screening practices [50].

In our study, 82.35% of diabetes cases were newly diagnosed, representing 6.3% of all participants. This rate surpasses the findings from similar studies such as 3.4% in Kenya [13] and 4.8% in rural South Africa [55]. Variations in lifestyles, inconsistent practices of HIV/NCD integrated services, diagnostic methods, NCD screening habits in ART clinics and limited self‐screening habits could have contributed to disparities across studies and settings. The high prevalence of T2DM may highlight significant gaps in routine screening practices within ART clinics in our study population.

The use of DTG‐based regimens was not associated with hyperglycaemia in this study. This finding is in line with the reports from previous studies that reported similar findings [22, 23]. In contrast, other reports suggest a potential association between the use of DTG‐based regimens and an increased risk of hyperglycaemia, possibly due to its effects on insulin sensitivity and glucose metabolism [8]. This inconsistency may result from variations in participants' demographics and potential interactions with lifestyle factors.

In this study, obesity was significantly associated with hyperglycaemia. This finding is consistent with the literature, which has demonstrated a robust association between altered glucose metabolism and increased weight [7, 16]. The weight gains among those who received the DTG‐based regimens [8] may exacerbate insulin resistance, compounded by synergistic metabolic effects with other ART medications and the presence of cardiometabolic problems, thereby contributing to dysglycaemia. Similarly, Rebeiro et al. [11] reported that initiating ART regimens with INSTIs rather than with NNRTIs may increase the risk of DM, potentially due to weight gain associated with INSTI use. In support of this, the majority of participants with hyperglycaemia (62.9%) and T2DM (64.7%) in our study had a BMI of ≥ 25 kg/m^2^. Moreover, adjusted analyses in this study also revealed a significant interaction between DTG‐based regimens and BMI in relation to hyperglycaemia, which may further support the results.

Our findings revealed an association between high WHR and hyperglycaemia, with the association of both elevated WHR and WC with T2DM. This finding is consistent with the reports of Njoroge et al., who highlighted the association between central adiposity and hyperglycaemia [13]. In support, elevated WHR and WC are indicative of central obesity that might impair insulin sensitivity and glucose metabolism, thereby increasing the risk of hyperglycaemia through inflammatory processes and hormonal disruption [56].

This study found a significant association between advanced age and hyperglycaemia. This is consistent with previous studies that have established an association between ageing and hyperglycaemia in PLWH [8, 57, 58]. Moreover, ageing might be linked to decreased insulin sensitivity due to physiological changes such as reduced muscle mass, impaired pancreatic β‐cell function and increased chronic inflammation, which disrupts insulin signalling and glucose metabolism [59].

In this study, we observed an association between hyperglycaemia and alcohol consumption. This finding aligns with previous studies indicating that alcohol intake may increase the risk of developing hyperglycaemia or diabetes [60, 61]. Additionally, a correlation between alcohol consumption and the incidence of T2DM has been reported in men [62]. The underlying mechanisms are complex and may involve alcohol's impact on insulin activity and glucose metabolism.

We found a significant association between LDL cholesterol and hyperglycaemia, as well as between triglycerides and T2DM. Similarly, several studies have shown a positive association between blood glucose levels and both triglycerides [63] and LDL cholesterol levels [63, 64]. In addition, elderly individuals with hypertriglyceridaemia, hypercholesterolaemia and low HDL cholesterol are at an increased risk of developing diabetes [65]. This may be attributed to the synergistic effects of insulin resistance and ART‐induced alterations in lipid metabolism, both of which contribute to the development and progression of hyperglycaemia and diabetes.

### 4.1 Limitations

This study has several limitations: primarily its cross‐sectional design, which restricts the ability to establish causal relationships between hyperglycaemia and its risk factors. The study focused on PLWH on ART without including HIV‐negative individuals as a reference group. However, comparisons were made with findings from prevously published studies in the general adult population, highlighting higher rates of hyperglycaemia among PLWH on ART. Additionally, most participants were on DTG‐based regimens, either through initiation or switching, resulting in an insufficient number of participants in the NNRTI‐based arm for comparison during analysis. Despite these limitations, this study may offer valuable insights into the limited data setting of East Africa, particularly in Ethiopia.

## 5 Conclusion

Our study found a higher prevalence of hyperglycaemia among PLWH compared to the general Ethiopian adult population, along with a significant rate of newly diagnosed T2DM. Modifiable risk factors contributing to hyperglycaemia included abdominal adiposity, BMI, dyslipidemia and alcohol intake. Non‐modifiable risks included ageing and the presence of other co‐morbidities with HIV. A significant interaction effect was observed between DTG‐based regimens and age > 50 years, as well as between DTG‐based regimens and BMI on hyperglycaemia.

The findings highlight potential gaps in routine screening and early detection of dysglycaemia in ART clinics, unless prompted by clients. Therefore, to improve early diagnosis and management, implementing routine screening protocols for all individuals on ART, regardless of symptoms, is highly recommended. Additionally, since most of the risk factors are modifiable, implementing targeted interventions focused on lifestyle and behavioural modifications for individuals with identified risk factors would be beneficial.

## Author Contributions

Agete Tadewos Hirigo, Zelalem Debebe, Daniel Yilma and Ayalew Astatkie conceptualised and designed the study; conducted the data analysis and drafted the initial manuscript; contributed to data interpretation, critically revised the manuscript for intellectual content and provided final approval for publication. Agete Tadewos Hirigo was responsible for data collection. Zelalem Debebe, Daniel Yilma and Ayalew Astatkie supervised the study. All authors reviewed and approved the final manuscript.

## Ethics Statement

The study protocol was approved by the Institutional Review Board of the College of Natural and Computational Sciences at Addis Ababa University (approval No: IRB/07/14/2022). The participants were assured the freedom to withdraw from the study at any time without repercussions. Participants were not exposed to any harm, and no experimental risks were present. Throughout the study, participants' personal information was maintained with strict confidentiality.

## Consent

The participants were fully informed about the research objectives and potential benefits, and then written consent was obtained from all participants.

## Conflicts of Interest

The authors declare no conflicts of interest.

## Supporting information


**Table S1.** Clinical and other characteristics of the study population.
**Table S2.** The interaction effect of antiretroviral regimens with other covariates on hyperglycaemia among the study population.
**Table S3.** Interaction effect of antiretroviral regimens with other covariates on hyperglycaemia among the study population stratified by sex.

## Data Availability

The data are not publicly accessible, but can be obtained upon reasonable request from the corresponding author.

## References

[1] P. Chhoun , C. Ngin , S. Tuot , et al., “Non‐Communicable Diseases and Related Risk Behaviors Among Men and Women Living With HIV in Cambodia: Findings From a Cross‐Sectional Study,” International Journal for Equity in Health 16, no. 1 (2017): 125. 28705242 10.1186/s12939-017-0622-y PMC5513209

[2] S. Zicari , L. Sessa , N. Cotugno , et al., “Immune Activation, Inflammation, and Non‐AIDS Co‐Morbidities in HIV‐Infected Patients Under Long‐Term ART,” Viruses 11, no. 3 (2019): 200. 30818749 10.3390/v11030200 PMC6466530

[3] A. I. Forray , M. A. Coman , R. Simonescu‐Colan , A. I. Mazga , R. M. Cherecheș , and C. M. Borzan , “The Global Burden of Type 2 Diabetes Attributable to Dietary Risks: Insights From the Global Burden of Disease Study 2019,” Nutrients 15, no. 21 (2023): 4613. 37960266 10.3390/nu15214613 PMC10648266

[4] M. R. Rooney , M. Fang , K. Ogurtsova , et al., “Global Prevalence of Prediabetes,” Diabetes Care 46, no. 7 (2023): 1388–1394. 37196350 10.2337/dc22-2376 PMC10442190

[5] N. Peer , K. A. Nguyen , J. Hill , et al., “Prevalence and Influences of Diabetes and Prediabetes Among Adults Living With HIV in Africa: A Systematic Review and Meta‐Analysis,” Journal of the International AIDS Society 26, no. 3 (2023): e26059. 36924213 10.1002/jia2.26059 PMC10018386

[6] N. Peer , K. Nguyen , J. Hill , and A.‐P. Kengne , “Prevalence of Diabetes and Prediabetes Among Adults With HIV in Africa: A Systematic Review and Meta‐Analysis,” Journal of Hypertension 40, no. Suppl 1 (2022): e187.

[7] M. Jemal , T. Shibabaw Molla , G. M. M. Tiruneh , E. Chekol Abebe , and T. Asmamaw Dejenie , “Blood Glucose Level and Serum Lipid Profiles Among People Living With HIV on Dolutegravir‐Based Versus Efavirenz‐Based cART; a Comparative Cross‐Sectional Study,” Annals of Medicine 55, no. 2 (2023): 2295435, 10.1080/07853890.2023.2295435. 38118463 PMC10763893

[8] L. H. Byereta , R. Olum , E. I. Mutebi , et al., “Prevalence and Factors Associated With Hyperglycemia Among Persons Living With HIV/AIDS on Dolutegravir‐Based Antiretroviral Therapy in Uganda,” Therapeutic Advances in Infectious Disease 11 (2024): 1272630. 10.1177/20499361241272630 PMC11403689 39286262

[9] F. Akello , D. Nalwanga , V. Musiime , and S. Kiguli , “Dolutegravir‐Related Hyperglycemia Among Children and Adolescents <18 Years in Northern and Eastern Uganda: A Cross‐Sectional Study,” medRxiv 12 (2023): 99497, 10.1101/2023.12.05.23299497.

[10] A. Mugoya and I. A. Ogwal , “Prevalence of Hyperglycemia in HIV Patients on Dolutegravir art Regimen Receiving Care at Jinja Regional Referral Hospital. A Cross‐Sectional Study,” SJ Diabetes, Hypertension and Cancer Research Africa 1, no. 6 (2024): 7.

[11] P. F. Rebeiro , C. A. Jenkins , A. Bian , et al., “Risk of Incident Diabetes Mellitus, Weight Gain, and Their Relationships With Integrase Inhibitor–Based Initial Antiretroviral Therapy Among Persons With Human Immunodeficiency Virus in the United States and Canada,” Clinical Infectious Diseases 73, no. 7 (2021): e2234–e2242. 32936919 10.1093/cid/ciaa1403 PMC8492142

[12] A. E. Mohammed , T. Y. Shenkute , and W. C. Gebisa , “Diabetes Mellitus and Risk Factors in Human Immunodeficiency Virus‐Infected Individuals at Jimma University Specialized Hospital, Southwest Ethiopia,” Diabetes, Metabolic Syndrome and Obesity: Targets and Therapy 8 (2015): 197–206. 25926749 10.2147/DMSO.S80084 PMC4403746

[13] A. Njoroge , O. Augusto , S. T. Page , et al., “Increased Risk of Prediabetes Among Virally Suppressed Adults With HIV in Central Kenya Detected Using Glycated Haemoglobin and Fasting Blood Glucose,” Endocrinology, Diabetes & Metabolism 4, no. 4 (2021): e00292. 10.1002/edm2.292 PMC8502220 34505404

[14] T. Fiseha and A. G. Belete , “Diabetes Mellitus and Its Associated Factors Among Human Immunodeficiency Virus‐Infected Patients on Anti‐Retroviral Therapy in Northeast Ethiopia,” BMC Research Notes 12, no. 1 (2019): 1–7. 31262341 10.1186/s13104-019-4402-1 PMC6604311

[15] K. Jeremiah , S. Filteau , D. Faurholt‐Jepsen , et al., “Diabetes Prevalence by HbA1c and Oral Glucose Tolerance Test Among HIV‐Infected and Uninfected Tanzanian Adults,” PLoS One 15, no. 4 (2020): e0230723. 32267855 10.1371/journal.pone.0230723 PMC7141607

[16] F. Duguma , W. Gebisa , A. Mamo , D. Tamiru , and S. Woyesa , “Diabetes Mellitus and Associated Factors Among Adult HIV Patients on Highly Active Anti‐Retroviral Treatment,” HIV/AIDS‐Research and Palliative Care 12 (2020): 657–665, 10.2147/HIV.S279732. 33162756 PMC7641867

[17] E. Biranu , M. Wolde , A. E. Negesso , M. M. Sisay , and H. H. Tola , “Lipid Profile, Abnormality of Serum Glucose Levels and Their Associated Factors in Multidrug‐Resistant Tuberculosis Patients,” Global Journal of Obesity, Diabetes and Metabolic Syndrome 8, no. 3 (2021): 18–28, 10.17352/2455-8583.000053.

[18] WHO. World Health Organization , Updated Recommendations on First‐Line and Second‐Line Antiretroviral Regimens and Post‐Exposure Prophylaxis and Recommendations on Early Infant Diagnosis of HIV: Interim Guidelines: Supplement to the 2016 Consolidated Guidelines on the Use of Antiretroviral Drugs for Treating and Preventing HIV Infection (World Health Organization, 2018).

[19] FMoH. Federal Ministry of Health of Ethiopia , “National Consolidated Guidelines for Comprehensive HIV Prevention, Care and Treatment.” (2018), https://www.humanitarianresponse.info/sites/www.humanitarianresponse.info/files/documents/files/national_comprehensive_hiv_care_guideline_2018‐endorsed.pdf.

[20] D. Namara , J. I. Schwartz , A. K. Tusubira , et al., “The Risk of Hyperglycemia Associated With Use of Dolutegravir Among Adults Living With HIV in Kampala, Uganda: A Case‐Control Study,” International Journal of STD & AIDS 33, no. 14 (2022): 1158–1164. 36222490 10.1177/09564624221129410 PMC9691558

[21] P. Kamal and S. Sharma , “SUN‐187 Dolutegravir Causing Diabetes,” Journal of the Endocrine Society 3, no. S1 (2019): SUN‐187.

[22] F. Mulindwa , H. Kamal , B. Castelnuovo , et al., “Association Between Integrase Strand Transfer Inhibitor Use With Insulin Resistance and Incident Diabetes Mellitus in Persons Living With HIV: A Systematic Review and Meta‐Analysis,” BMJ Open Diabetes Research & Care 11, no. 1 (2023): e003136. 10.1136/bmjdrc-2022-003136 PMC9923267 36754450

[23] V. D. Kajogoo , W. Amogne , and G. Medhin , “New Onset Type 2 Diabetes Mellitus Risks With Integrase Strand Transfer Inhibitors‐Based Regimens: A Systematic Review and Meta‐Analysis,” Metabolism Open 17 (2023): 100235. 36923992 10.1016/j.metop.2023.100235 PMC10009287

[24] P. Patel , C. Speight , A. Maida , et al., “Integrating HIV and Hypertension Management in Low‐Resource Settings: Lessons From Malawi,” PLoS Medicine 15, no. 3 (2018): e1002523. 29513674 10.1371/journal.pmed.1002523 PMC5841643

[25] L. G. Hemkens and H. C. Bucher , “HIV Infection and Cardiovascular Disease,” European Heart Journal 35, no. 21 (2014): 1373–1381. 24408888 10.1093/eurheartj/eht528

[26] D. M. Belay , W. A. Bayih , A. Y. Alemu , et al., “Diabetes Mellitus Among Adults on Highly Active Anti‐Retroviral Therapy and Its Associated Factors in Ethiopia: Systematic Review and Meta‐Analysis,” Diabetes Research and Clinical Practice 182 (2021): 109125, 10.1016/j.diabres.2021.109125. 34742783

[27] M. Moyo‐Chilufya , K. Maluleke , K. Kgarosi , M. Muyoyeta , C. Hongoro , and A. Musekiwa , “The Burden of Non‐Communicable Diseases Among People Living With HIV in Sub‐Saharan Africa: A Systematic Review and Meta‐Analysis,” eClinicalMedicine 65 (2023): 102255. 37842552 10.1016/j.eclinm.2023.102255 PMC10570719

[28] Committee ADAPP , “Committee: ADAPP. 2. Classification and Diagnosis of Diabetes: Standards of Medical Care in Diabetes—2022,” Diabetes Care 45, no. S1 (2022): S17–S38. 34964875 10.2337/dc22-S002

[29] W. Walelgne , D. Yadeta , Y. Feleke , and T. Kebede , Guidelines on Clinical and Programmatic Management of Major Non Communicable Diseases, vol. 1 (Federal Democratic Republic of Ethiopia Ministry of Health, 2016), 15–25.

[30] Omron , Full Body Sensor Body Composition Monitor and Scale (OMRON HBF‐514C) (Omron Healthcare Co. Ltd), (2021).

[31] Organization WH , “Waist Circumference and Waist‐Hip Ratio: Report of a WHO Expert Consultation, Geneva, 8‐11 December 2008.” (2011).

[32] C. Serviente and G. A. Sforzo , “A Simple Yet Complicated Tool: Measuring Waist Circumference to Determine Cardiometabolic Risk,” ACSM's Health & Fitness Journal 17, no. 6 (2013): 29–34, 10.1249/FIT.0b013e3182a956f5.

[33] M. Ashwell and S. Gibson , “Waist to Height Ratio Is a Simple and Effective Obesity Screening Tool for Cardiovascular Risk Factors: Analysis of Data From the British National Diet and Nutrition Survey of Adults Aged 19–64 Years,” Obesity Facts 2, no. 2 (2009): 97–103. 20054212 10.1159/000203363 PMC6444829

[34] A. Paini , C. Aggiusti , F. Bertacchini , et al., “Office Blood Pressure Measurement: Mean of Two or of Three Values?,” Journal of Hypertension 40, no. Suppl 1 (2022): e93–e94, 10.1097/01.hjh.0000836140.50714.69.

[35] C. L. Craig , A. L. Marshall , M. Sjöström , et al., “International Physical Activity Questionnaire: 12‐Country Reliability and Validity,” Medicine & Science in Sports & Exercise 35, no. 8 (2003): 1381–1395, 10.1249/01.MSS.0000078924.61453.FB. 12900694

[36] WHO , “World Health Organization. Promoting Fruit and Vegetable Consumption.” (2004), http://www.euro.who.int/en/health‐topics/disease‐prevention/nutrition/activities/technical‐support‐to‐member‐states/promoting‐fruit‐and‐vegetable‐consumption.

[37] K. Meressa , A. Mossie , and Y. Gelaw , “Effect of Substance Use on Academic Achievement of Health Officer and Medical Students of Jimma University, Southwest Ethiopia,” Ethiopian Journal of Health Sciences 19, no. 3 (2009): 155–163.

[38] A. Montazeri , M. Vahdaninia , M. Ebrahimi , and S. Jarvandi , “The Hospital Anxiety and Depression Scale (HADS): Translation and Validation Study of the Iranian Version,” Health and Quality of Life Outcomes 1, no. 1 (2003): 1–5, 10.1186/1477-7525-1-14. 12816545 PMC161819

[39] F. Ambaw , “The Structure and Reliability of the Amharic Version of the Hospital Anxiety and Depression Scale in Orphan Adolescents in Addis Ababa,” Ethiopian Journal of Health Sciences 21, no. 1 (2011): 27–36. 22434983 10.4314/ejhs.v21i1.69041 PMC3275850

[40] M. Dibbasey , S. Umukoro , and A. Bojang , “Comparative and Stability Study of Glucose Concentrations Measured in Both Sodium Fluoride and Serum Separator Tubes,” Practical Laboratory Medicine 39 (2024): e00360. 38313813 10.1016/j.plabm.2024.e00360 PMC10832486

[41] A. Al‐Kharusi , N. Al‐Lawati , M. Al‐Kindi , and W.‐A. Mula‐Abed , “Are Tubes Containing Sodium Fluoride Still Needed for the Measurement of Blood Glucose in Hospital Laboratory Practice?,” Oman Medical Journal 29, no. 6 (2014): 404–407. 25584156 10.5001/omj.2014.109 PMC4289486

[42] L. Fernandez , P. Jee , M. J. Klein , P. Fischer , S. L. Perkins , and S. P. Brooks , “A Comparison of Glucose Concentration in Paired Specimens Collected in Serum Separator and Fluoride/Potassium Oxalate Blood Collection Tubes Under Survey ‘field’ Conditions,” Clinical Biochemistry 46, no. 4–5 (2013): 285–288, 10.1016/j.clinbiochem.2012.11.027. 23219741

[43] M. Bhargava , N. P. Singh , and A. K. Gupta , “Should We Still Collect Blood Glucose Sampling in Fluoride Tubes? An Evidence‐Based Study,” International Journal of Diabetes in Developing Countries 39 (2019): 243–244.

[44] C. Pheiffer , V. Pillay‐van Wyk , E. Turawa , N. Levitt , A. P. Kengne , and D. Bradshaw , “Prevalence of Type 2 Diabetes in South Africa: A Systematic Review and Meta‐Analysis,” International Journal of Environmental Research and Public Health 18, no. 11 (2021): 5868. 34070714 10.3390/ijerph18115868 PMC8199430

[45] M. Abebe , S. Kinde , G. Belay , et al., “Antiretroviral Treatment Associated Hyperglycemia and Dyslipidemia Among HIV Infected Patients at Burayu Health Center, Addis Ababa, Ethiopia: A Cross‐Sectional Comparative Study,” BMC Research Notes 7 (2014): 380. 24950924 10.1186/1756-0500-7-380 PMC4077831

[46] M. A. Abera , M. H. Tequare , E. Berhe , A. L. Wolderufael , H. E. Abraha , and M. M. Ebrahim , “Dolutegravir‐Associated Hyperglycemia in People Living With Human Immune‐Deficiency Virus: A Prospective Cohort Study.” (2022).

[47] Z. Ataro , W. Ashenafi , J. Fayera , and T. Abdosh , “Magnitude and Associated Factors of Diabetes Mellitus and Hypertension Among Adult HIV‐Positive Individuals Receiving Highly Active Antiretroviral Therapy at Jugal Hospital, Harar, Ethiopia,” HIV/AIDS‐Research and Palliative Care 10 (2018): 181–192. 30349400 10.2147/HIV.S176877 PMC6190641

[48] P. Shankalala , C. Jacobs , S. Bosomprah , M. Vinikoor , P. Katayamoyo , and C. Michelo , “Risk Factors for Impaired Fasting Glucose or Diabetes Among HIV Infected Patients on ART in the Copperbelt Province of Zambia,” Journal of Diabetes & Metabolic Disorders 16 (2017): 1–8. 28725640 10.1186/s40200-017-0310-x PMC5513349

[49] T. Borkowska , N. Chkhartishvili , E. Karkashadze , et al., “The Prevalence of Hyperglycemia and Its Impact on Mortality Among People Living With HIV in Georgia,” PLoS One 17, no. 10 (2022): e0276749. 36301817 10.1371/journal.pone.0276749 PMC9612544

[50] F. Bahamdain , “Effect of Dolutegravir on Plasma Glucose Among Human Immunodeficiency Virus Patients in a Community Health Center Setting,” Cureus 14, no. 10 (2022): e30556, 10.7759/cureus.30556. 36303801 PMC9586418

[51] Y. F. Gebreyes , D. Y. Goshu , T. K. Geletew , et al., “Prevalence of High Bloodpressure, Hyperglycemia, Dyslipidemia, Metabolic Syndrome and Their Determinants in Ethiopia: Evidences From the National NCDs STEPS Survey, 2015,” PLoS One 13, no. 5 (2018): e0194819. 29742131 10.1371/journal.pone.0194819 PMC5942803

[52] A. D. Duncan , L. M. Goff , and B. S. Peters , “Type 2 Diabetes Prevalence and Its Risk Factors in HIV: A Cross‐Sectional Study,” PLoS One 13, no. 3 (2018): e0194199. 29529066 10.1371/journal.pone.0194199 PMC5847234

[53] H. Sun , P. Saeedi , S. Karuranga , et al., “IDF Diabetes Atlas: Global, Regional and Country‐Level Diabetes Prevalence Estimates for 2021 and Projections for 2045,” Diabetes Research and Clinical Practice 183 (2022): 109119. 34879977 10.1016/j.diabres.2021.109119 PMC11057359

[54] G. Roglic and Organization WH , Global Report on Diabetes (World Health Organization, 2016) 2018.

[55] A. E. de Vries , Z. Xaba , S. R. Moraba , et al., “Unmasking a Silent Killer: Prevalence of Diagnosed and Undiagnosed Diabetes Mellitus Among People Living With HIV in Rural South Africa,” Tropical Medicine & International Health 28, no. 5 (2023): 367–373, 10.1111/tmi.13871. 36920286

[56] M. S. Burhans , D. K. Hagman , J. N. Kuzma , K. A. Schmidt , and M. Kratz , “Contribution of Adipose Tissue Inflammation to the Development of Type 2 Diabetes Mellitus,” Comprehensive Physiology 9, no. 1 (2018): 1–58. 30549014 10.1002/cphy.c170040 PMC6557583

[57] C. GLumer , B. Carstensen , A. Sandbæk , T. Lauritzen , T. Jørgensen , and K. Borch‐Johnsen , “A Danish Diabetes Risk Score for Targeted Screening: The Inter99 Study,” Diabetes Care 27, no. 3 (2004): 727–733, 10.2337/diacare.27.3.727. 14988293

[58] L. D. Rasmussen , E. R. Mathiesen , G. Kronborg , C. Pedersen , J. Gerstoft , and N. Obel , “Risk of Diabetes Mellitus in Persons With and Without HIV: A Danish Nationwide Population‐Based Cohort Study,” PLoS One 7, no. 9 (2012): e44575, 10.1371/journal.pone.0044575. 22984529 PMC3440341

[59] P. G. Lee and J. B. Halter , “The Pathophysiology of Hyperglycemia in Older Adults: Clinical Considerations,” Diabetes Care 40, no. 4 (2017): 444–452. 28325795 10.2337/dc16-1732

[60] N. Dereje , A. Earsido , L. Temam , and A. Abebe , “Prevalence and Associated Factors of Diabetes Mellitus in Hosanna Town, Southern Ethiopia,” Annals of Global Health 86, no. 1 (2020): 18, 10.5334/aogh.2663. 32140428 PMC7047764

[61] C. Cao , C. Wei , Y. Han , et al., “Association Between Excessive Alcohol Consumption and Incident Diabetes Mellitus Among Japanese Based on Propensity Score Matching,” Scientific Reports 14, no. 1 (2024): 17274. 39068183 10.1038/s41598-024-68202-3 PMC11283479

[62] J. Song and W.‐Q. Lin , “Association Between Alcohol Consumption and Incidence of Type 2 Diabetes Mellitus in Japanese Men: A Secondary Analysis of a Retrospective Cohort Study,” BMC Endocrine Disorders 23, no. 1 (2023): 91. 37098575 10.1186/s12902-023-01350-1 PMC10127320

[63] L. Wang , N. Yan , M. Zhang , R. Pan , Y. Dang , and Y. Niu , “The Association Between Blood Glucose Levels and Lipids or Lipid Ratios in Type 2 Diabetes Patients: A Cross‐Sectional Study,” Frontiers in Endocrinology 13 (2022): 969080. 36147575 10.3389/fendo.2022.969080 PMC9485560

[64] E. Ndagijimana , A. Nshimiyimana , T. Habyarimana , C. Izere , and F. N. Niyonzima , “Association of Blood Glucose and Lipid Profile Concentrations in Diabetic Patients Attending Gisenyi District Hospital in Rwanda: A Cross‐Sectional Study,” East Africa Science 6, no. 1 (2024): 62–69.

[65] J. Peng , F. Zhao , X. Yang , et al., “Association Between Dyslipidemia and Risk of Type 2 Diabetes Mellitus in Middle‐Aged and Older Chinese Adults: A Secondary Analysis of a Nationwide Cohort,” BMJ Open 11, no. 5 (2021): e042821. 10.1136/bmjopen-2020-042821 PMC8154929 34035089

