# Supplementary material for: Hyperglycaemia and Its Risk Factors Among Adults Living With HIV on Follow‐Up at the Hawassa City Administration, Southern Ethiopia: A Cross‐Sectional Study
Source: Endocrinol Diabetes Metab. 2025 Apr 27;8(3):e70054. doi: 10.1002/edm2.70054 (PMC12034571; doi:10.1002/edm2.70054)
Supplement: Supplementary file 1 — Table S1. Clinical and other characteristics of the study population. Table S2. The interaction effect of antiretroviral regimens with other covariates on hyperglycaemia among the study population. Table S3. Interaction effect of antiretroviral regimens with other covariates on hyperglycaemia among the study population stratified by sex. [file EDM2-8-e70054-s001.docx]

**Table S1** Clinical and other characteristics of the study population

| Variables | Category |  | Variables | Category |  |
| --- | --- | --- | --- | --- | --- |
| BMI, kg/m^2^ | Mean(SD) | 24.7(4.8) | Vegetables intake | <4days/week | 355(80.1%) |
| BMI category | ≤18.4 kg/m^2^ | 27(6.1%) |  | ≥4days/week | 88(19.9%) |
|  | 18.5-24.9 kg/m^2^ | 220(49.7%) | Physical activity | Inadequate | 143(32.3%) |
|  | 25-29.9 kg/m^2^ | 138(31.2%) |  | Moderate | 244(55.1%) |
|  | ≥30 kg/m^2^ | 58(13.1%) |  | Highly active | 56(12.6%) |
| HIV duration, years | Median(IQR) | 4.2(2.3-6.1) | Intake of meat | ≤3days/week | 420(94.8%) |
| ART duration, years | Mean (SD) | 3.8(1.9) |  | ≥4days/week | 23(5.2%) |
| Smoking status | Never | 379(85.6%) | Family history of  DM | Don’t know | 23(5.2%) |
|  | Former | 54(12.2%) |  | No | 337(76.1%) |
|  | Current | 10(2.3%) |  | Yes | 83(18.7%) |
| Alcohol intake status | Never | 185(41.8%) | WC, cm | Median(IQR) | 85(76-94) |
|  | Former | 172(38.8%) | HC, cm | Median(IQR) | 95(89-102) |
|  | Current | 86(19.4%) | WHR | Median(IQR) | 0.9(0.83-0.96) |
| Khat chewing status | Never | 276(62.3%) | WHtR | Median(IQR) | 0.52(0.47-0.6) |
|  | Former | 132(29.8%) | TGs, mg/dL | Median(IQR | 119(87.9-175) |
|  | Current | 35(7.9%) | TC, mg/dL | Mean(SD) | 159.3(37.3) |
| Comorbidity with HIV | No | 357(80.6%) | LDL-c, mg/dL | Mean(SD) | 91.1(30.9) |
|  | Yes | 86(19.4%) | HDL-c, mg/dL | Mean(SD) | 36.6(9.6) |
| Fruit intake | <4days/week | 391(88.3%) | SBP, mmHg | Median(IQR) | 117(107-128) |
|  | ≥4days/week | 52(11.7%) | DBP, mmHg | Mean(SD) | 77.4(12.5) |
| ART, antiretroviral therapy; BMI, body mass index; DBP, diastolic blood pressure; DM, diabetes mellitus; kg, kilogram; m^2^, square meter; LDL-c, LDL-cholesterol; HC, hip circumference; HDL-c, HDL-cholesterol; IQR, interquartile range; mg/dL, milligram/deciliter; mmHg, millimeter of mercury; SBP; systolic blood pressure; SD, standard deviation; TC, total cholesterol; TGs, triglycerides; WC, waist circumference; WHR, waist-hip ratio; WHtR, waist-height ratio | | | | | |

**Table S2** The interaction effect of antiretroviral regimens with other covariates on hyperglycemia among the study population

| Interaction parameters | Treatment category | Hyperglycaemia | |
| --- | --- | --- | --- |
|  |  | Adjusted odds ratio (95%CI) | p value |
| Treatment groups  on male sex | Efavirenz-maintained | reference |  |
|  | Switched to dolutegravir | 1.4(0.75-2.7) | 0.278 |
|  | Dolutegravir-maintained | 0.98(0.52-1.8) | 0.945 |
| Treatment groups  on age >50years | Efavirenz-maintained | reference |  |
|  | Switched to dolutegravir | 1.9(0.84-4.3) | 0.122 |
|  | Dolutegravir-maintained | 2.4(1.03-5.7) | 0.041 |
| Treatment groups on BMI 25-29.9 kg/m^2^ | Efavirenz-maintained | reference |  |
|  | Switched to dolutegravir | 2.3(1.2-4.3) | <0.001 |
|  | Dolutegravir-maintained | 1.7(0.86-3.2) | 0.127 |
| Treatment groups on BMI ≥30 kg/m^2^ | Efavirenz-maintained | reference |  |
|  | Switched to dolutegravir | 4.8(2.1-11.1) | <0.001 |
|  | Dolutegravir-maintained | 2.9(1.2-6.8) | 0.017 |

BMI, body mass index; CI, confidence interval; kg, kilogram; m^2^, square meter; **Reference category:** female; age ≤50 years; BMI <25kg/m^2^

**Table S3** Interaction effect of antiretroviral regimens with other covariates on hyperglycemia among the study population stratified by sex

| Interaction variables | Treatment category | Hyperglycaemia | |
| --- | --- | --- | --- |
|  |  | Adjusted odds ratio (95%CI) | p value |
| **Women**  Treatment groups on age >50years | Efavirenz-maintained | reference |  |
|  | Switched to dolutegravir | 2.3(0.62-8.4) | 0.218 |
|  | Dolutegravir-maintained | 4.4(1.1-17.6) | 0.034 |
| Treatment groups on TGs ≥150 mg/dL | Efavirenz-maintained | reference |  |
|  | Switched to dolutegravir | 2.7(1.1-6.5) | 0.029 |
|  | Dolutegravir-maintained | 3.5(1.4-9.0) | 0.008 |
| Treatment groups on BMI 25-29.9 kg/m^2^ | Efavirenz-maintained | reference |  |
|  | Switched to dolutegravir | 2.9(1.2-7.1) | 0.018 |
|  | Dolutegravir-maintained | 4.7(1.7-13.3) | 0.003 |
| Treatment groups on BMI ≥30 kg/m^2^ | Efavirenz-maintained | reference |  |
|  | Switched to dolutegravir | 1.6(0.57-4.3) | 0.38 |
|  | Dolutegravir-maintained | 2.1(0.68-6.5) | 0.198 |
| **Men**  Treatment groups on TGs ≥150 mg/dL | Efavirenz-maintained | reference |  |
|  | Switched to dolutegravir | 1.4(0.53-3.8) | 0.479 |
|  | Dolutegravir-maintained | 0.86(0.29-2.6) | 0.788 |
| Treatment regimens on age ≥50years | Efavirenz-maintained | reference |  |
|  | Switched to dolutegravir | 1.6(0.55-4.5) | 0.45 |
|  | Dolutegravir-maintained | 1.4(0.46-4.1) | 0.57 |
| Treatment groups on BMI 25-29.9 kg/m^2^ | Efavirenz-maintained | reference |  |
|  | Switched to dolutegravir | 1.5(0.5-4.6) | 0.462 |
|  | Dolutegravir-maintained | 15.8(1.7-145.1) | 0.015 |
| Treatment groups on BMI ≥30 kg/m^2^ | Efavirenz-maintained | reference |  |
|  | Switched to dolutegravir | 1.5(0.48-5.0) | 0.468 |
|  | Dolutegravir-maintained | 3.8(0.50-29.5) | 0.194 |

BMI, body mass index; CI, confidence interval; kg, kilogram; m^2^, square meter; TGs, triglycerides; **Reference category**: age ≤50 years; TGs <150 mg/dL; BMI <25 kg/m^2^
